# Abscisic Acid Induces Adventitious Rooting in Cucumber (*Cucumis sativus* L.) by Enhancing Sugar Synthesis

**DOI:** 10.3390/plants11182354

**Published:** 2022-09-09

**Authors:** Changxia Li, Meiling Zhang, Nana Qi, Huwei Liu, Zongxi Zhao, Panpan Huang, Weibiao Liao

**Affiliations:** 1 College of Horticulture, Gansu Agricultural University, 1 Yinmen Village, Anning District, Lanzhou 730070, China; 2 College of Science, Gansu Agricultural University, Lanzhou 730070, China

**Keywords:** cucumber, adventitious root formation, abscisic acid, sugar synthesis

## Abstract

Abscisic acid (ABA) affects many important plant processes, such as seed germination, root elongation and stomatal movement. However, little information is available about the relationship between ABA and sugar synthesis during adventitious root formation. The aim of this study was to evaluate the effect of ABA on adventitious root formation in cucumber and whether the effect of this plant hormone on sugar synthesis could be included as a causative factor for adventitious root development. We determined the contents of glucose, sucrose, starch, total sugar and sugar-related enzymes, including sucrose synthase (SS), sucrose phosphate synthase (SPS), hexokinase (HK) and pyruvate kinase (PK) activities in ABA treatment. We also quantified the relative expression of sucrose or glucose synthesis genes during this process. Increasing ABA concentrations significantly improved adventitious root formation, with the most considerable effect at 0.05 μM. Compared to the control, ABA treatment showed higher glucose, sucrose, starch and total sugar contents. Moreover, ABA treatment increased glucose-6-phosphate (G6P), fructose-6-phosphate (F6P) and glucose-1-phosphate (G1P) contents in cucumber explants during adventitious root development, which was followed by an increase of activities of sucrose-related enzymes SS and SPS, glucose-related enzymes HK and PK. ABA, meanwhile, upregulated the expression levels of sucrose or glucose synthesis-related genes, including *CsSuSy1*, *CsSuSy6*, *CsHK1* and *CsHK3*. These results suggest that ABA may promote adventitious root development by increasing the contents of glucose, sucrose, starch, total sugar, G6P, F6P and G1P, the activities of SS, SPS, HK, SPS and the expression levels of *CsSuSy1*, *CsSuSy6*, *CsHK1* and *CsHK3* genes. These findings provide evidence for the physiological role of ABA during adventitious root formation and provide a new understanding of the possible relationship between ABA and sugar synthesis during adventitious rooting in plants.

## 1. Introduction

Abscisic acid (ABA), as one of the plant hormones, is considered an important signaling molecule. ABA occupies an enviable place in mediating plant development and growth, such as seed germination, seed dormancy, embryo development, cell division and elongation, floral induction and stomatal movement [1,2,3,4]. Rapid advances in the area of ABA signaling have helped shed light on plant developmental processes. ABA is also involved in plant response to a series of environmental stresses, such as drought, salt and temperature stresses [5]. Importantly, Chin et al. [6] indicated that ABA stimulated the rooting of the stem cuttings of mung beans and English ivy. It has also been found that ABA may stimulate adventitious root initiation in mung bean *Vigna radiata* cuttings [7]. However, exogenous ABA inhibited peanut seedlings’ lateral root development by increasing endogenous ABA content [8]. ABA also acts as an inhibitor of lateral root development in *Arachis hypogaea* by blocking cell cycle progression [9]. Inhibition of the adventitious root formation step by ABA was also reported in deep-water rice [10]. Similarly, shoot-derived ABA inhibits lateral root development and adventitious root formation in tomato, likely via effects on ethylene (ET) and indoleacetic acid (IAA) pathways [11]. ABA regulates root growth and development through crosstalk with IAA and cytokinins (CTK) in *Brassica napus* L. [12]. Dong et al. [13] indicated that ABA-induced expression 1 (AIN1) was involved in the ABA-mediated inhibition of root elongation by modulating ROS homeostasis.

Sugars can be converted into polymers, giving rise to storage components such as starch and fructans or structural components such as cellulose. Sugar sensing and signaling are crucial to balance the requirement of nutrient, hormone and environmental signals in plants. Cytosolic triose phosphates are substrates for sucrose synthesis in which fructose-1,6-bisphosphatase (F6P) and sucrose phosphate synthase (SPS) have been identified as rate-limiting [14,15]. In plants, sucrose, as the majority abundant sugar, may be transported to sink organs or cleaved by invertases to glucose and fructose [16]. Glucose, recognized as a central signaling molecule, plays an important regulatory role, as well as a universal carbon and energy source. Hexokinase (HK) is an evolutionarily conserved glucose sensor in a wide range of organisms, including plants [17]. HK, as the first enzyme in glycolysis, phosphorylates glucose to glucose-6-phosphate (G6P). Both hexokinase-dependent and hexokinase-independent glucose signal transduction pathways appear to coexist [18]. Sugar may influence diverse plant growth and development processes, such as seed germination, development, photosynthesis, flowering and senescence [19]. Meanwhile, sugar is also involved in various abiotic stresses, including water stress, salt stress and temperature stress [20,21,22]. Several reports have shown that sugar influences plant root growth. For example, in *Arabidopsis*, sucrose promotes lateral root formation [23] and adventitious root initiation under dark conditions [24]. Mishra et al. [25] found that increasing glucose concentrations significantly increased root length and the number of lateral roots and root hairs. Glucose has also been shown to regulate lateral root formation via G-protein signaling [26]. Exogenous application of glucose to 7-day-old *Arabidopsis* seedlings for 3 days repressed primary root growth by shortening the root meristematic zone [27]. Sucrose as a source of energy was involved in adventitious root regeneration in apple [28].

Although ABA and sugar regulate adventitious rooting, the potential mechanism of ABA in sugar synthesis during adventitious rooting in cucumber remains unknown. To the best of our knowledge, this study is the first report on ABA in sugar synthesis during adventitious rooting and further investigates the signal regulatory network of adventitious rooting in plants.

## 2. Materials and Methods

### 2.1. Plant Material

Cucumber (*Cucumis sativus* ‘Xinchun NO. 4′) seeds were surface sterilized for 10 min in 5% (*w*/*v*) sodium hypochlorite and then soaked with distilled water for 6 h. Then, the seeds were germinated on filter paper containing distilled water in Petri dishes (15-cm diameter, 2.5 cm deep) and transferred to an illuminating incubator at 25 ± 1 °C for 6 days with a 14-h photoperiod (photosynthetically active radiation 200 μmol s^−1^m^−2^). Seedlings with primary roots removed were used as explants. Then, the cucumber explants were maintained under the same temperature and photoperiod conditions described above for another 5 days in the presence of different media, as indicated below. The number, length and fresh weight of adventitious roots per explant were then measured and recorded. In addition, the explants were cultivated under the same temperature and photoperiod conditions described above for another 2 days in the presence of different media indicated below. Finally, 1 cm long segments of the hypocotyl base (Figure S1) were taken as the experimental material and used for the following analysis.

### 2.2. Explant Treatments

The explants were cultivated at various concentrations of ABA (0, 0.01, 0.02, 0.05, 0.10 and 1.00 μM; Sigma, St. Louis, MO, USA). The ABA concentration used was closely related to the experimental materials and experimental conditions. Some studies have suggested that the ABA concentration used in different issues is different [29,30]. Additionally, under stress conditions, cucumber explants were treated with 0.5 μM ABA that was reported by Li et al. [31]. Our experiment was carried out under normal conditions. In our pre-experiment, when ABA was greater than 1 μM such as 2, 5 and 10 μM, it caused the explants to lose water, wilt and die. Thus, a low dose of ABA (less than 1 μM) was used in this study. The following chemicals were carried out: control (distilled water) and 0.05 μM ABA. For each treatment, three replicates of cucumber plants were performed. The concentration of these chemicals was selected based on the results of a preliminary experiment.

### 2.3. Determination of Glucose Content

Glucose was analyzed as described by Leach et al. [32], with some modifications. Briefly, 0.5 g of the sample was homogenized with 10 mL distilled water, diluted with distilled water to 50 mL, and mixed. The volumetric flask was then placed in a warm-water bath at 50 °C for 10 min. The homogenate was centrifuged at 4000× *g* rpm at 4 °C for 15 min. A 3,5-dinitrosalicylic acid (DNS) regent was prepared in advance. First, 6.3 g of 3,5-dinitrosalicylic acid was dissolved in 262 mL of a 2 M sodium hydroxide solution. Then, 185 g of potassium sodium tartrate was added to 500 mL of hot water solution and mixed until it dissolved fully. Lastly, the two solutions were mixed, followed by adding 5 g of redistilled phenol and 5 g of sodium sulfite, mixed and cooled to room temperature, and the volume was brought up to 1000 mL with distilled water. The supernatant (2 mL) and 1.5 mL of DNS were mixed together. Then, it was placed in a boiling water bath for 5 min, rapidly cooled and then filtered into a 25 mL volumetric flask. Absorbance at 540 nm was recorded using a spectrophotometer (UV-1601, Shimadzu, Kyoto, Japan).

### 2.4. Determination of Sucrose Content

The sucrose content was measured according to Wu et al. [33], with minor modifications. The sample (1.0 g) was homogenized with 10 mL of 80% ethanol and diluted to 100 mL. The volumetric flask was placed in a water bath at 80 °C for 45 min, rapidly cooled and then filtered. The filtrate was collected as the reaction mixture. The reaction mixture (0.4 mL) and 0.2 mL of 2 M NaOH were mixed together, placed in a water bath at 80 °C for 10 min, and rapidly cooled. The absorbance at 480 nm was recorded using a spectrophotometer (UV-1601).

### 2.5. Determination of Starch Content

The method described by Hou et al. [34] was adopted to determine the starch content. The frozen sample (0.5 g) was ground in 2 mL of distilled water, and then 3.2 mL of 60% HClO_4_ was added. After blending, the solution was transferred to a tube and diluted to 10 mL. The mixture was centrifuged at 5000× *g* r/min for 5 min. The supernatant (0.5 mL) was diluted to 3 mL with distilled water, and 2 mL of iodine reagent was added. After the reaction for 5 min, the mixture was diluted to 10 mL with distilled water. The absorbance was measured at 660 nm using a spectrophotometer (UV-1601).

### 2.6. Determination of Total Sugar Content

The total sugar content was determined by the procedures described by Zhao et al. [35]. One gram of sample tissue was homogenized in 10 mL of 6 M HCl and diluted to 25 mL with distilled water. The test tube was placed in a boiling water bath for 30 min to hydrolyze the sugar. Using phenolphthalein as an indicator, the total sugar content was determined with 6 M NaOH’s standard titration solution. Then, the mixture was diluted to 100 mL with distilled water, mixed well and filtered. The filtrate (10 mL) was transferred to the new test tube, diluted to 100 mL with distilled water, and analyzed. Absorbance at 540 nm was performed using a spectrophotometer (UV-1601).

### 2.7. G6P, F6P and G1P Content Measurements

The enzyme was extracted using the modified method of Wei et al. [36]. Briefly, 0.5 g of the samples were grounded in liquid nitrogen. Then, it was added to 2 mL of 5% trichloroacetic acid (TCA) consisting of 100 mg polyvinyl polypyrrolidone (PVPP). The mixtures were centrifuged at 12,000× *g* rpm for 15 min at 4 °C. The supernatant was collected, and 150 μL of neutralizing buffer containing 1 M triethanolamine and 5 M potassium hydroxide (KOH) was added to the supernatant. After a reaction for 30 min on ice, the mixture was centrifuged for 15 min at 12,000× *g* r/min. The supernatants were collected as enzyme extracts for further analysis.

For G6P, F6P and glucose-1-phosphate (G1P), the reaction mixture consisted of 497 μL of distilled water, 100 μL of 1 M Hepes-KOH (pH 7.6), 100 μL of 50 mM MgCl_2_, 100 μL 4 mM NAD, 100 μL of 10 mM EDTA and 100 μL of crude enzyme extract. The formation of blue formazan was monitored by recording the absorbance at 560 nm. G6PDH, PGI and PGM were added successively for G6P, F6P and G1P mixtures, and then were determined by measuring the absorbance at 340 nm using a spectrophotometer (UV-1601).

### 2.8. SS, SPS, HK and PK Enzyme Activity Measurement

Enzyme extracts were performed as described previously [37]. Samples were harvested, ground in liquid nitrogen, and homogenized in 9 mL of ice-cold PBS (pH = 7.4). Then, extracts were centrifuged at 3000× *g* rpm for 15 min at 4 °C. The activities of sucrose synthase (SS), sucrose phosphate synthase (SPS), hexokinase (HK) and pyruvate kinase (PK) enzyme were measured by enzyme-linked immunosorbent assay (ELISA; AndyGene Biotechnology Co. Ltd., Beijing, China) according to the manufacturer’s instructions.

### 2.9. Quantitative Real-Time PCR (qRT-PCR)

Total RNA was extracted using TRIzol (Invitrogen Life Technologies) according to the method described by Huang et al. [38]. RNA was reverse transcribed with the 5 × *Evo M-MLV*RT Master Mix (AG, Changsha, China) according to the manufacturer’s instructions. The cDNA was amplified with 2 × SYBR Green *Pro Taq* HS Premix (AG, China) using the following primers shown in Table 1. The reactions were controlled by the following conditions: 30 s at 95 °C, then 5 s at 95 °C and 30 s at 60 °C for 40 cycles. Quantitative real-time PCR reactions were performed with a Mastercycler eprealplex real-time PCR system (Eppendorf, Hamburg, Germany). The expression levels of *CsSuSy1*, *CsSuSy6*, *CsHK1* and *CsHK3* genes were presented as values relative to the corresponding control samples under the indicated conditions, with normalization of data to the geometric average of internal cucumber *TUA* [39]. The expression level of the gene was calculated by 2^−ΔΔCT^. ΔCT = CT (target gene) − CT (internal reference gene). ΔΔCT = ΔCT (test group) − ΔCT (control group). The relative expression level of the gene = 2^−ΔΔCT^. Each sample was set to three biological replicates.

### 2.10. Data Statistics and Analysis

Where indicated, the results were expressed as the mean values ± standard error (SE) of at least three independent experiments. Statistical analysis was performed using SPSS 22.0. For statistical analysis, a t-test (*p* < 0.05) was chosen.

## 3. Results

### 3.1. Effect of Exogenous ABA on Adventitious Root Development

To understand the effects of exogenous ABA on adventitious rooting in cucumber, explants were treated with increasing concentrations of ABA (Table 2). In comparison with the control, ABA had a significant effect on cucumber adventitious rooting. Among the different concentrations of ABA, the maximum root number, root length and fresh weight were observed for 0.05 μM ABA (Table 2). Thus, treatment with 0.05 μM ABA was used for further studies.

### 3.2. Effect of Exogenous ABA on Glucose, Sucrose, Starch and Total Sugar Contents during Adventitious Rooting

**Figure 1:**
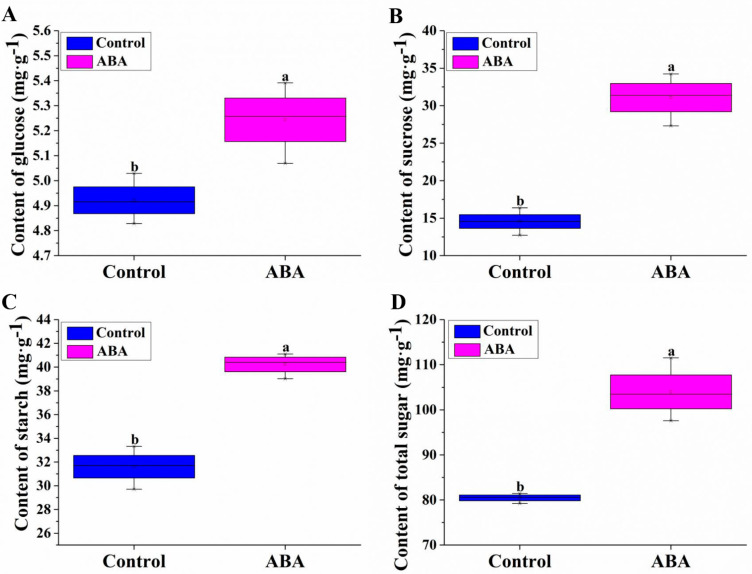
Effects of exogenous 0.05 μM ABA on glucose (**A**), sucrose (**B**), starch (**C**) and total sugar (**D**) contents in hypocotyls of cucumber. The values (means ± SE) are the averages of the three independent experiments. The different letters above the bars indicate significant differences (*p* < 0.05) according to one-way ANOVA and *t* test.

The effects of ABA on glucose, sucrose, starch and total sugar contents during adventitious root formation were investigated. Compared to the control, the glucose, sucrose, starch and total sugar contents in ABA-treated hypocotyls were significantly increased, by approximately 6.54%, 113.61%, 27.29% and 29.29%, respectively (Figure 1). Therefore, our results suggested that ABA treatment significantly increased the contents of glucose, sucrose, starch and total sugar.

### 3.3. Effect of Exogenous ABA on G6P, F6P and G1P Contents during Adventitious Root Development

**Figure 2:**
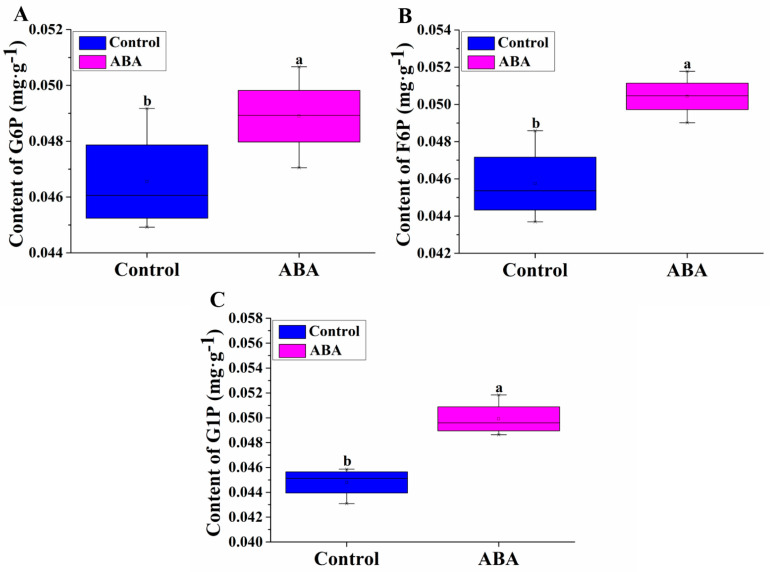
Effects of exogenous 0.05 μM ABA on G6P (**A**), F6P (**B**) and G1P (**C**) contents in hypocotyls of cucumber. The values (means ± SE) are the average concentrations of the three independent experiments. The different letters above the bars indicate significant differences (*p* < 0.05) according to one-way ANOVA and *t* test. G6P, glucose-6-phosphate; F6P, fructose-6-phosphate; G1P, glucose-1-phosphate.

We next measured the contents of 3 hexose phosphates in hypocotyls treated with ABA to detect the effect of sugar synthesis during adventitious rooting. The G6P content in the hypocotyls treated with ABA was significantly higher than that in the control (Figure 2A). Meanwhile, ABA significantly increased the F6P and G1P content by approximately 10.20% and 14.86%, respectively (Figure 2B,C).

### 3.4. Effect of Exogenous ABA on SS, SPS, HK and PK Activities during Adventitious Rooting

**Figure 3:**
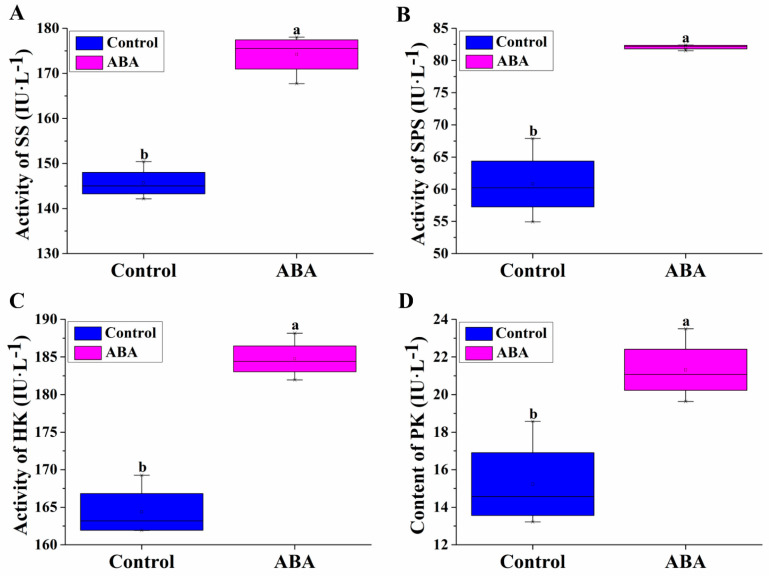
Effects of exogenous 0.05 μM ABA on SS (**A**), SPS (**B**), HK (**C**) and PK (**D**) enzyme activities in hypocotyls of cucumber. The values (means ± SE) are the averages of the three independent experiments. The different letters above the bars indicate significant differences (*p* < 0.05) according to one-way ANOVA and *t* test. IU/L: Activity of the enzyme solution per liter. SS, sucrose synthase; SPS, sucrose phosphate synthase; HK, hexokinase; PK, pyruvate kinase.

To examine the promotion roles of ABA in SS, SPS, HK and PK activities in detail, we determined the activities of SS, SPS, HK and PK in ABA-treated/untreated hypocotyls. ABA treatment significantly enhanced the SS, SPS, HK and PK activities with respect to the control by increasing 19.61%, 34.97%, 16.38% and 39.96%, respectively (Figure 3). These data revealed that ABA regulated the SS, SPS, HK and PK activities in the sugar synthesis pathway.

### 3.5. Effect of Exogenous ABA on CsSuSy1, CsSuSy6, CsHK1 and CsHK3 Expression Levels

**Figure 4:**
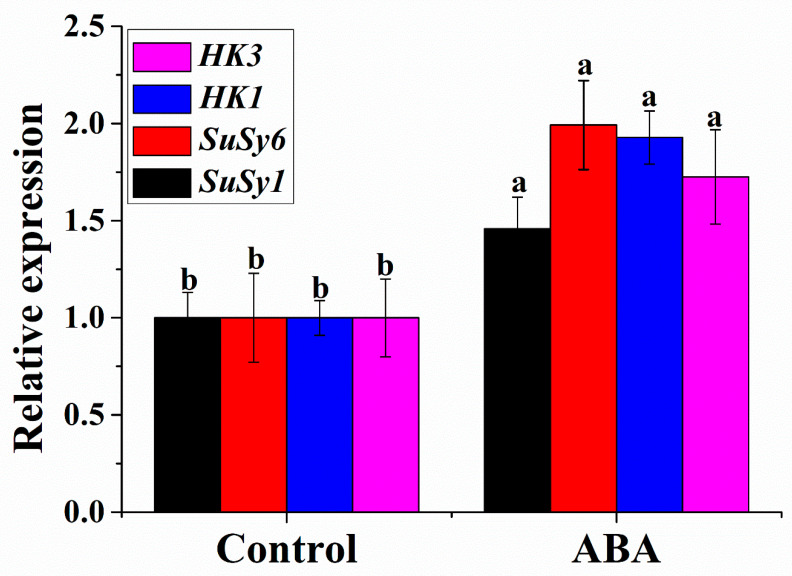
Effects of exogenous 0.05 μM ABA on the expression levels of *CsSuSy1*, *CsSuSy6*, *CsHK1* and *CsHK3 genes* in hypocotyls of cucumber. The values (means ± SE) are the averages of three independent experiments. The different letters above the bars indicate significant differences (*p* < 0.05) according to one-way ANOVA and *t* test.

To gain insight into the sugar synthesis-related mechanism of ABA-induced adventitious root development, the relative expression of related genes, *CsSuSy1*, *CsSuSy6*, *CsHK1* and *CsHK3,* was analyzed by qRT-PCR. Compared with the control, ABA treatment was able to induce higher expression of the *CsSuSy1*, *CsSuSy6*, *CsHK1* and *CsHK3* genes during adventitious root formation, increased by 1.46, 1.99, 1.73, and 1.93-fold, respectively (Figure 4).

## 4. Discussion

The importance of ABA in plant root systems has been extensively recognized. ABA took part in the regulation of rice root system growth under simulated acid rain stress [40]. Li et al. [41] reported that pretreatment with ABA promoted the formation and growth of adventitious roots in *mung* bean under cadmium stress. Geng et al. [42] found that a suitable ABA concentration promoted root development, but high concentrations of ABA inhibited root growth. Li et al. [31] found that ABA significantly enhanced adventitious root formation in cucumbers under drought stress. Research has also shown that ABA repressed ethylene-induced and gibberellin-promoted emergence of adventitious roots in deepwater rice [10]. ABA is also involved in the inhibition of primary root elongation in rice and wheat under normal/non-stress conditions [43,44,45]. In the study, different ABA concentrations promoted adventitious rooting, and 0.05 μM ABA had the most effective (Table 2). Given the above, ABA might regulate adventitious root development in cucumber. However, our knowledge of the cross-talk between ABA and sugar signaling during adventitious rooting is unclear.

Sucrose is the major sugar translocated in plants and can be cleaved by invertase to glucose and fructose. Starch is an insoluble polymer of glucose produced by most higher plant species. The interaction between ABA and sugar signaling during early seedling development in Arabidopsis has been revealed [46]. ABA is a positive regulator of sucrose accumulation in citrus and grape [47]. Exogenous ABA can accelerate glucose and fructose accumulation [48]. Pan et al. [49] reported that ABA promoted sugar synthesis and accumulation in grape berry. Sucrose combined with ABA synergistically regulated the expression levels of 15 starch biosynthetic genes in maize endosperm [50]. The expression levels of *AtAPL3* and *OsAPL3* genes were upregulated in the sucrose-treated Arabidopsis leaves and cultured rice cells, respectively, resulting in an increase in starch content [51,52]. Additionally, the expression levels of the two genes were further enhanced in the sucrose plus ABA treatment. Huang et al. [53] reported that ZmEREB156 positively modulated the starch biosynthetic gene *ZmSSIIIa* through the synergistic effect of sucrose and ABA. In this study, the glucose, sucrose, starch and total sugar contents under ABA treatment were significantly increased compared to the control (Figure 1). Starch is degraded to G1P by phosphorylation. ABA promoted glucose, sucrose, starch and total sugar accumulations, followed by increasing G6P, F6P and G1P contents (Figure 2). These results indicate that ABA may significantly promote glucose, sucrose, starch and total sugar accumulations by increasing G6P, F6P and G1P contents.

There is a massive transformation of starch to sugars during plant growth and development, in which several enzymes are included, such as SPS and SS. Shi et al. [37] reported that SPS and SS, as key glucose synthesis enzymes, were responsible for sucrose accumulation in longan fruit. SS activity was also induced by sucrose and ABA in rice [54]. Tan et al. [48] also indicated that ABA increased the activities of SS and SPS in longan fruit. We also found that ABA significantly increased SS and SPS activities during adventitious root development. These data show that ABA increased sucrose content during cucumber adventitious rooting by enhancing SS and SPS activities (Figure 3). This might be one reason for sucrose accumulation. Hexokinase (HK) and pyruvate kinase (PK) are the key enzymes responsible for the glucose and fructose synthesis pathways. In the present study, the exposure of cucumber hypocotyls to ABA resulted in an increase in the activities of HK and PK enzymes, suggesting that ABA may be involved in glucose synthesis by increasing HK and PK activities during adventitious root formation.

Sugars have a signaling function in which the HK and SuSy proteins are suggested to play a pivotal role. To test the conclusion further, the expression levels of *CsSuSy1*, *CsSuSy6*, *CsHK1* and *CsHK3* with or without ABA were analyzed. ABA treatment induced higher expression levels of *CsSuSy1*, *CsSuSy6*, *CsHK1* and *CsHK3* genes than the control (Figure 4). Dominguez et al. [55] reported that ABA imparted on glucose signaling mediated by *HK1. HK1* participates in the hypocotyl elongation of Arabidopsis promoted by glucose under dark conditions [56]. The combination of ABA and sucrose had synergic effects on the sucrose induction of the anthocyanin synthesis pathway [57]. Jia et al. [58] reported that abscisic acid-stress-ripening (ASR) was involved in the transduction of ABA and sucrose signaling pathways to regulate tomato and strawberry fruit ripening. Thus, the results have important functional implications, expanding and enriching the possibilities for ABA during adventitious root development.

## 5. Conclusions

In summary, this study demonstrated that ABA treatment promoted adventitious root development in the cucumber. ABA-treated cucumber hypocotyls also showed higher glucose, sucrose, starch and total sugar content than samples from untreated ABA. Moreover, ABA treatment increased G6P, F6P and G1P contents, which was followed by the increase of sucrose-related enzymes SS and SPS and glucose-related enzymes HK and PK activities. Meanwhile, the relative expression of sucrose and glucose synthesis-related genes, including *CsSuSy1*, *CsSuSy6*, *CsHK1* and *CsHK3,* were significantly upregulated. Taken together, these results indicate that ABA treatment regulates the adventitious rooting of cucumber and that sugar synthesis may be regulated by ABA during this process.

## Figures and Tables

**Table 1:** Sequences of primers used for qRT-PCR analysis.

Gene Symbol	Accession Number	Primer Sequence (5′-3′)
*CsSuSy1-F* *CsSuSy1-R* *CsSuSy6-F* *CsSuSy6-R* *CsHK3-F* *CsHK3-R* *CsHK1-F* *CsHK1-R*	LOC101213767 LOC101216865 LOC101218300 LOC101215511	CGTGTGCTAAGGAAGGCGGAAG CAGTGTCACCCCACCCTCTCTC TCCAACCGCCACAACTTCATCAC CCATTCCCACTCTGCCCAAGC CACGGTCCTAGTCAGTCGGAGAG GCCATAGCATCAACCACCTGTCTC CGCCATGACCGTCGAGATGC TTTGTACCGCCGAGATCCAATGC
*CsTUA-F* *CsTUA-R*	AJ715498	ACGCTGTTGGTGGTGGTAC GAGAGGGGTAAACAGTGAATC

**Table 2:** Effect of ABA on adventitious root development in cucumber.

ABA/μM	Root Number	Root Length (mm)	Fresh Weight (g)
0.00	3.21 ± 032 d	6.16 ± 0.23 d	0.1708 ± 0.0036 c
0.01	4.75 ± 0.52 c	8.04 ± 0.25 c	0.2025 ± 0.0054 b
0.02	5.29 ± 0.40 b	8.39 ± 0.42 b	0.2104 ± 0.0064 b
0.05	7.62 ± 0.37 a	10.55 ± 0.04 a	0.2460 ± 0.0017 a
0.10	4.71 ± 0.25 b	7.69 ± 0.35 c	0.2225 ± 0.0034 b
1.00	1.41 ± 0.22 e	4.79 ± 0.31 e	0.1610 ± 0.0013 d

The different letters indicate significant differences (*p* < 0.05). *n* = 18 explants from each of three independent experiments were carried out.

## Data Availability

Not applicable.

## Supplementary Materials

The following supporting information can be downloaded at: https://www.mdpi.com/article/10.3390/plants11182354/s1, Figure S1. Patterns of cucumber explants and experimental materials.
