# Supplementary figures and images for: Abscisic Acid Induces Adventitious Rooting in Cucumber (Cucumis sativus L.) by Enhancing Sugar Synthesis
Source: Plants (Basel). 2022 Sep 9;11(18):2354. doi: 10.3390/plants11182354 (PMC9505232; doi:10.3390/plants11182354)

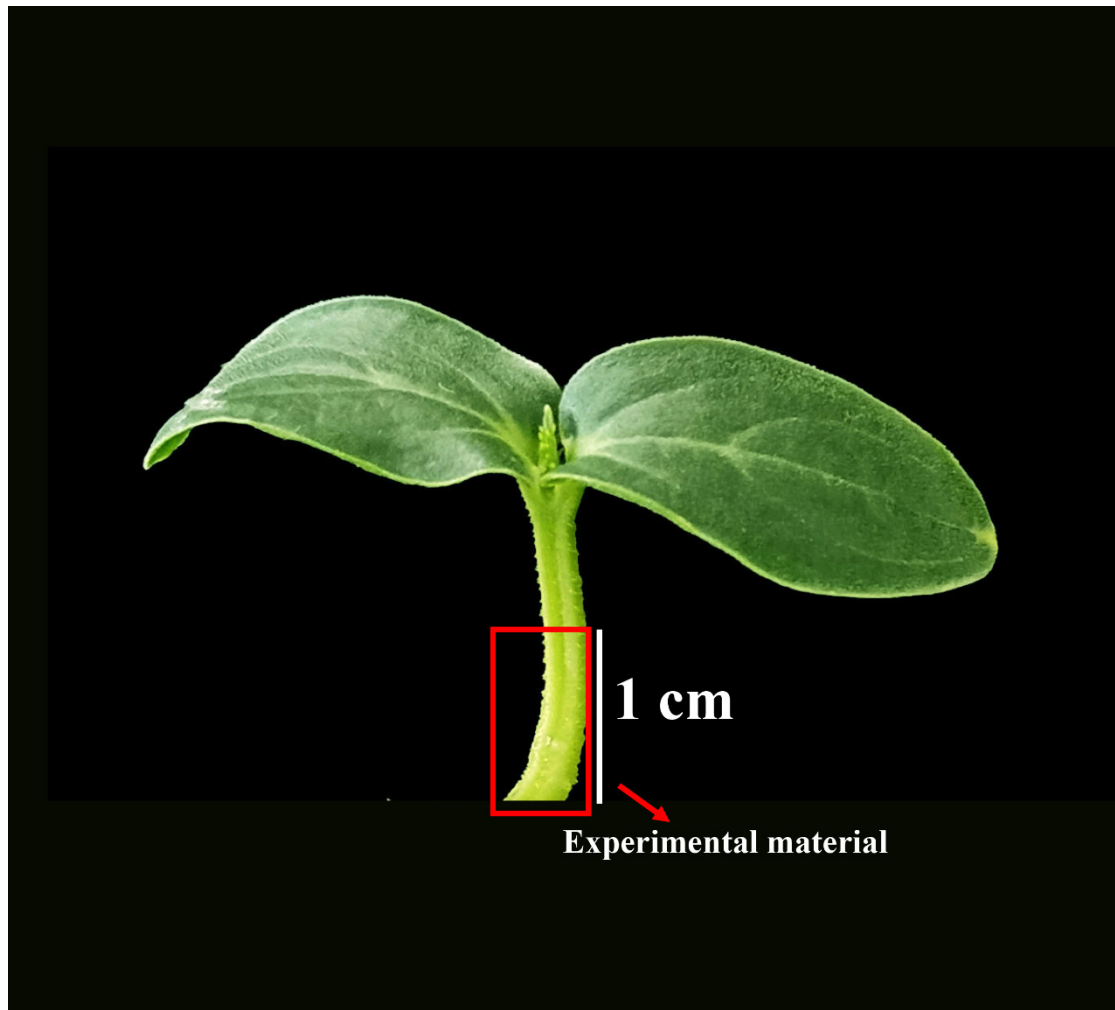

**Figure S1.** Patterns of cucumber explants and experimental materials

Supplement: Supplementary file 1 [file plants-11-02354-s001.zip › plants-1789927-supplementary.pdf]
